# Molecular Characterization of Influenza A(H3N2) Hemagglutinin Variants Circulating in Western Mexico, 2022

**DOI:** 10.3390/ijms27156833

**Published:** 2026-07-30

**Authors:** Karen M Hernandez-Gonzalez, Ahtziri Socorro Carranza-Aranda, José Francisco Muñoz-Valle, Luis Alfonso Muñoz-Miranda, Alejandra Natali Vega-Magaña, Ana Laura Pereira-Suárez, Cesar Arturo Nava-Valdivia

**Affiliations:** 1 Programa de Doctorado en Microbiología Médica, Departamento de Microbiología y Patología, Centro Universitario de Ciencias de la Salud (CUCS), Universidad de Guadalajara, Guadalajara 44340, Jalisco, Mexico; karen.hernandez1070@alumnos.udg.mx; 2 Departamento de Disciplinas Filosóficas, Metodológicas e Instrumentales, Centro Universitario de Ciencias de la Salud (CUCS), Universidad de Guadalajara, Guadalajara 44340, Jalisco, Mexico; 3 Instituto de Investigación en Ciencias Biomédicas, Centro Universitario de Ciencias de la Salud (CUCS), Universidad de Guadalajara, Guadalajara 44340, Jalisco, Mexico; 4 Centro de Investigación en Enfermedades Infectocontagiosas, Departamento de Microbiología y Patología, Centro Universitario de Ciencias de la Salud (CUCS), Universidad de Guadalajara, Guadalajara 44340, Jalisco, Mexico; 5 Instituto de Investigación en Cáncer e Infecciones, Centro Universitario de Ciencias de la Salud (CUCS), Universidad de Guadalajara, Guadalajara 44340, Jalisco, Mexico; 6 Laboratorio de Diagnóstico en Enfermedades Emergentes y Reemergentes (LaDEER), Centro Universitario de Ciencias de la Salud (CUCS), Universidad de Guadalajara, Guadalajara 44340, Jalisco, Mexico

**Keywords:** influenza A(H3N2), hemagglutinin, genetic variants, molecular characterization, receptor-binding domain, molecular docking

## Abstract

Influenza A(H3N2) remains a significant public health threat due to its rapid antigenic drift, which often compromises vaccine effectiveness. This study characterized the molecular and epidemiological profile of hemagglutinin (HA) variants circulating in Western Mexico throughout 2022. Among 476 positive cases, A(H3N2) was the predominant subtype (88%), with infection peaks during epidemiological weeks (EW) 1, 45, and 46. Sanger sequencing of the HA gene identified 64 amino acid substitutions, with 85.9% of the substitutions located in the HA1 subunit, primarily within the receptor-binding domain (RBD). Homology modeling and molecular docking were performed on five representative variants: C156S, D158N, Y159N, C136S, and L227P. All variants exhibited a slight decrease in binding affinity for sialic acid compared to the 1HGE reference. Notably, while mutations such as D158N and Y159N remodeled the interaction network, Glu190 and His183 remained critical for stabilizing the HA-ligand complex through hydrogen bonds and non-covalent interactions in the structural models. These structural findings suggest that contemporary mutations in the RBD may contribute to changes in receptor-binding interactions while preserving key structural features associated with host cell attachment.

## 1. Introduction

Influenza viruses remain a major global public health concern, causing recurrent seasonal epidemics and sporadic pandemics associated with substantial morbidity and mortality worldwide. Among influenza virus A subtypes, A(H3N2) is especially relevant because its rapid antigenic drift drives frequent outbreaks and reduces vaccine effectiveness. It represents a substantial burden of illness, particularly in vulnerable populations such as older adults, young children, and immunocompromised patients. Infection typically manifests with fever, cough, sore throat, myalgia, and malaise, but can also progress to severe complications including pneumonia and acute respiratory distress syndrome [1].

Seasonal influenza causes an estimated 3–5 million cases of severe illness and approximately 290,000–650,000 deaths worldwide each year, making it a persistent global health threat [2]. In Mexico, influenza A(H3N2) has been one of the predominant circulating strains during multiple seasonal outbreaks [3]. During the 2019–2020 influenza season, A(H3N2) accounted for approximately 19% of all influenza A cases Following the relaxation of public health measures related to COVID-19, 2022 marked the first period of widespread resurgence of influenza A(H3N2), characterized by increased viral circulation and the predominance of this subtype. This epidemiological scenario highlighted the need to characterize viral circulation due to the scarcity of molecular surveillance data from Western Mexico during this period [4,5].

This reduced efficacy is largely because immunization primarily elicits antibodies targeting the viral surface glycoproteins hemagglutinin (HA) and neuraminidase (NA). These proteins are essential for the viral life cycle: HA mediates viral attachment to host cell receptors, and NA facilitates the release of progeny virions from infected cells. Because of their critical functions and the strong selective pressure imposed by the host immune response, both HA and NA are highly prone to mutation [6]. However, the continuous remodeling of the RBD within HA has increasingly compromised this approach. The rapid diversification of A(H3N2) viruses, coupled with altered receptor-binding properties, has made the antigenic characterization of circulating strains progressively more challenging, complicating the selection of accurate vaccine strains [7].

At the structural level, HA is the most abundant glycoprotein on the influenza virus envelope and is a homotrimer made up of two glycopeptides, HA1 and HA2. Structurally, the trimer is organized into a globular head domain (primarily HA1) and a membrane-proximal stem domain (mainly HA2). Within HA1, the fusion (F), vestigial esterase domains (E), and RBD are found, while HA2 contains the membrane-proximal fusion and membrane-anchoring regions. As the main antigen of influenza viruses, the globular head of H3 HA has five principal antigenic sites (A–E), which are highly variable and responsible for antigenic drift [8,9].

Among these, antigenic site B has recently drawn attention as the immunodominant region of H3 HA. Due to its proximity to the RBD, it is functionally constrained, and mutations in this area can simultaneously change antigenicity and receptor affinity, thereby impacting both immune escape and viral transmissibility [10].

The RBD itself consists of four structural elements: the 130-loop, 150-loop, 190-helix, and 220-loop. It recognizes sialylated glycans on host cell glycoproteins and glycolipids, with host specificity determined by glycan linkage. Avian influenza viruses bind *α*2,3-linked sialic acids, whereas human viruses preferentially recognize *α*2,6 linkages. Therefore, adaptation of avian HA is necessary for sustained human transmission. Since its emergence in 1968, the HA of Influenza A(H3N2) has undergone substantial evolutionary changes in the RBD. Early strains favored short *α*2,6-linked glycans, but subsequent variants, including N158 and N159, introduced additional glycan contacts, increasing affinity for extended glycans and enhancing viral fitness [9,11].

Given the intricate structural remodeling of the HA RBD, computational approaches such as molecular docking have become central tools for quantifying the biophysical consequences of antigenic drift. By estimating relative binding free energies (Δ*G*) and atomic-level interactions between HA trimers and diverse sialylated glycans, docking provides a high-resolution framework to map evolving receptor preferences and infer potential host-range shifts. This in silico approach is particularly useful for evaluating how substitutions at positions 158, 159, 193, and within the 190-helix remodel hydrogen-bond networks and van der Waals contacts across the 130- and 190-loop elements. Furthermore, docking supports the structure-based design of HA-targeted antivirals and the modeling of antibody–epitope interfaces, helping to connect genomic surveillance with functional assessments of viral fitness and immune escape [12,13].

## 2. Results

### 2.1. Epidemiological and Demographic Characterization

**Figure 1:**
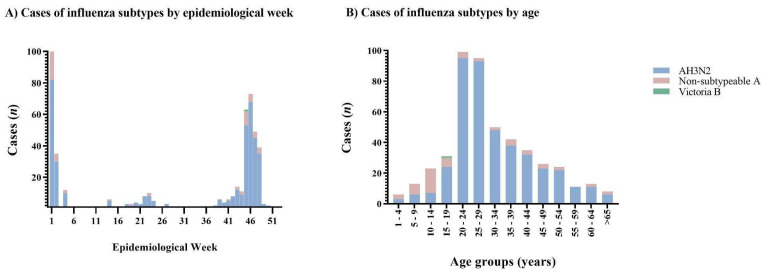
Cases of influenza subtypes by epidemiological week during 2022. (**A**) Bimodal pattern of influenza activity was observed during 2022, with major peaks occurring during epidemiological weeks 1–3 and 40–48. A/H3N2 was the predominant subtype throughout the year and accounted for most cases during both epidemic waves, whereas non-subtypeable influenza A viruses were detected less frequently and followed a similar temporal distribution. Influenza B viruses of the Victoria lineage were rarely identified, with only a single case detected in epidemiological week 45. Influenza activity remained low during the intervening weeks, with only sporadic cases reported. (**B**) By age group, the highest number of cases occurred among individuals aged 15–24 years, followed by those aged 25–29 years, with a progressive decline in older age groups. A/H3N2 predominated across all age categories.

During the study period in 2022, a total of 476 influenza cases were analyzed, including 251 females (52.7%) and 225 males (47.3%). The most prevalent subtype was influenza A(H3N2), accounting for 88% (*n* = 419) of the infections. This was followed by non-subtypeable influenza A at 11.8% (*n* = 56), and a minor presence of influenza B lineage Victoria, representing only 0.2% (*n* = 1) of the total cases. The distribution by EW revealed a high seasonal variability (Figure 1A). The primary peak occurred in EW 1, with 100 reported cases (21% of the total annual cases), where 82% (*n* = 82) were identified as A(H3N2) and 18% (*n* = 18) as non-subtypeable influenza A. A second significant increase in activity was observed in EW 46, recording 73 cases, characterized by a high predominance of A(H3N2) at 93.2% (*n* = 68). The third-highest peak was documented in EW 45, with 63 cases (13.2%), comprising 84.1% (*n* = 53) A(H3N2), 14.3% (*n* = 9) non-subtypeable A, and 1.6% (*n* = 1) influenza B/Victoria.

Regarding sex distribution, A(H3N2) was the predominant strain in both groups, affecting 89.2% (*n* = 224) of women and 86.7% (*n* = 195) of men. Non-subtypeable influenza A accounted for 10.4% (*n* = 26) and 13.3% (*n* = 30) of cases in women and men, respectively. A single case of influenza B/Victoria (0.4%) was reported in a female patient.

No statistically significant differences were observed in the distribution of viral subtypes between sexes (Chi-square test, *p* = 0.391).

Analysis by age group revealed that the highest frequency of influenza infections occurred in young adults aged 20–24 years, where A(H3N2) was the predominant subtype (96%, *n* = 95), followed by non-subtypeable influenza A (4%, *n* = 4). A similar trend was observed in the 25–29 and 30–34 age groups, with A(H3N2) representing over 96% of cases in both cohorts. In contrast, the extremes of the age spectrum showed lower case frequencies; the 1–4 age group presented three cases of A(H3N2) and three cases of non-subtypeable influenza A, while individuals over 65 years accounted for six A(H3N2) cases and two non-subtypeable A cases (Figure 1).

### 2.2. Phylogenetic Analysis of the HA Fragment

**Figure 2:**
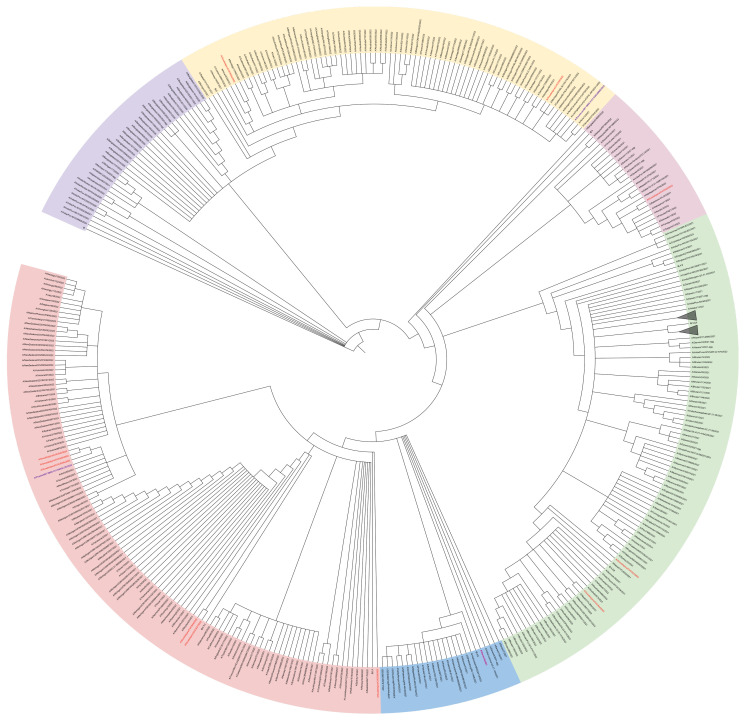
Phylogenetic tree of influenza HA gene segments. Subclades are color-coded as follows: clade G (violet), clade G2 (yellow), clade G1 (mauve), clades G1.3, G1.3.1, and G1.3.2 (green), clade G1.2 (blue), and clades G.1.1 and G.1.1.1 (pink). HA sequences are shown in red. Vaccine strain (A/Darwin/6/2022) is highlighted in blue and Mexican HA sequences in purple (A/Zacatecas/IBT IMSS/2022 and A/Puebla/IBT IMSS/2022).

Out of the total influenza-positive samples, 12 were partially sequenced. Phylogenetic analysis of the HA gene was conducted using Nextclade to characterize the circulating A(H3N2) variants. The sequenced HA samples clustered within subclades G.1 (*n* = 1, 8.3%), G.1.1 (*n* = 4, 33.3%), G.1.1.1 (*n* = 3, 25.0%), G.1.3.2 (*n* = 2, 16.7%), and G.2 (*n* = 2, 16.7%), which correspond to the clades 3C.2a1b.2a.2a, 3C.2a1b.2a.2a.1, 3C.2a1b.2a.2a.1a, 3C.2a1b.2a.2a.3b, and 3C.2a1b.2a.2b, respectively. These groups included reference sequences from 2021 to 2023 originating from North America, Asia, Europe, and North Africa. Notably, two Mexican sequences (A/Zacatecas/IBT IMSS/2022 and A/Puebla/IBT IMSS/2022) were identified within these subclade clusters, appearing in close proximity to the A/Darwin/6/2021 vaccine strain (Figure 2).

### 2.3. Identification of Variants in HA Sites

From these partial sequences, amino acid substitutions were analyzed, focusing exclusively on the region corresponding to the hemagglutinin gene. A total of 64 amino acid substitutions were identified in the HA protein (Supplementary Table S1). The highest number of variants was observed in week 45 (*n* = 11, 17.2%), followed by week 24 (*n* = 9, 14.1%), and week 23 (*n* = 8, 12.5%). The week with the fewest variants was week 39, with two variants (3.1%), followed by weeks 22, 28, 48, and 50, each with four variants detected (6.3%).

**Figure 3:**
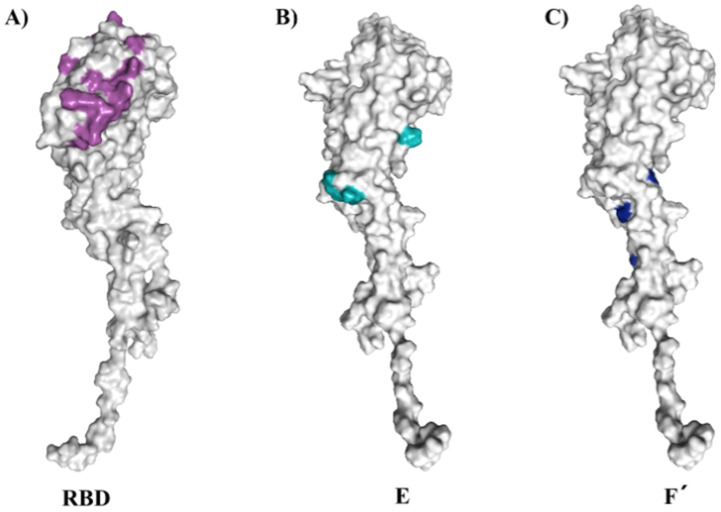
Location of mutations in the hemagglutinin of the H3N2 virus. (**A**) 32 point mutations from C136 to N262 in the RBD region (purple); (**B**) 6 point mutations at G86, D275, and M277 in region E (light blue); (**C**) 6 point mutations from E286 to E315 in region F’ (navy blue).

Overall, 55 variants (85.9%) were observed in the HA1 protein region, in contrast with the HA2 region where a total of 9 (14.1%) changes were detected (Supplementary Table S1). Within the HA1 subunit, the subdomain with the highest number of variants was the RBD, with 39 changes (60.9%), followed by the E region with 10 substitutions (15.6%) and the F’ region with 6 changes (9.4%). In the HA2 subunit, a total of 4 variants were identified in the F-HR fusion peptide region (6.3%), followed by the transmembrane domain with 3 changes (4.7%) and the linker region with 2 changes (3.1%) (Figure 3).

### 2.4. Structural Modeling of HA Variants

The sequences utilized for homology modeling exhibited a high degree of similarity (98.5–99%) with the reference structures. Consequently, these sequence-level variations exerted a moderate impact on the final protein conformation, particularly within the RBD spanning residues 136 to 227 (Supplementary Figure S2 and Table 1).

Structural alterations observed in models V1-C156S and V4-C136S involved the substitution of small amino acid residues with chemically similar ones (C → S). In contrast, models V2-D158N, V3-Y159N, and V5-L227P featured substitutions with residues of greater structural complexity or different physicochemical properties (D → N; L → P), which may potentially modify the binding site architecture and specific molecular interactions with sialic acid.

The five generated HA models demonstrated high structural quality according to ERRAT, VERIFY 3D, QMEAN, MolProbity, ProSA, and ProQ indices. The V6-all model (reference) displayed the highest stability scores, followed by models V2-D158N, V5-L227P, V1-C156S, V4-C136S, and V3-Y159N (Table 1).

The reference values used by each program to determine the structural quality of models are as follows: ERRAT considers a structure to be of high quality when the val-ues are equal to or greater than 95% (high resolution: 1.5–2 Å), while values below 91% correspond to lower resolution and structural quality (2.5–3 Å). VERIFY 3D determines that an optimal structure must at least 80% of amino acid score ≥ 0.1. QMEAN ana-lyzes structural quality based on individual residual errors and overall structural er-rors; values close to 0 indicate high structural quality, while very negative values (−4.0) reflect a high number of structural errors. Molprobity determines that values below 2 are indicative of high structural quality, based on the geometric scores of the Rama-chandran plots. ProSA determines a Z-score value based on structural quality by comparing the total energy of the structure with the energy of experimentally determined random conformations. Significantly negative Z-score values indicate high structural quality and stability, while positive values reflect conformational errors or energetically unfavorable conformations. ProQ predicts the overall quality of models based on the frequency of atomic contacts. On this scale, scores above 3.0 or 5.0 indicate good and excellent quality, respectively.

**Figure 4:**
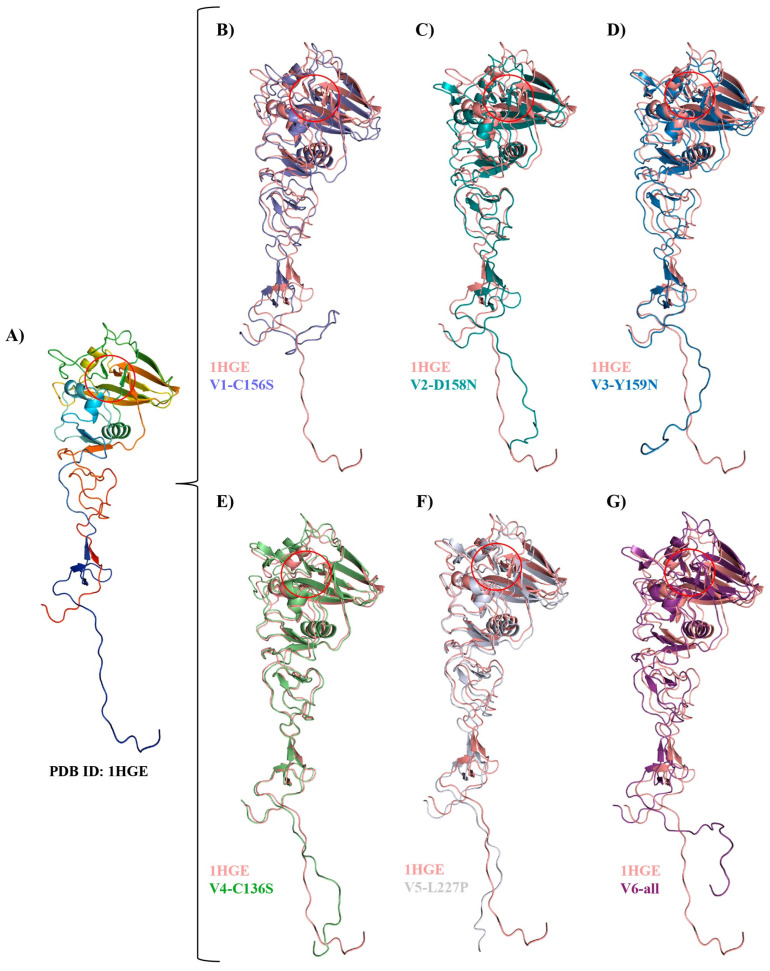
Structural comparison of crystallized hemagglutinin (PDB ID: 1HGE) versus models of H3N2 hemagglutinin variants. (**A**) Structure of HA (PDB ID: 1HGE); (**B**) Structure of HA variant 1 (C156S) (violet color); (**C**) Structure of HA variant 2 (D158N) (aqua blue color); (**D**) Structure of HA variant 3 (Y159N) (bright blue color); (**E**) Structure of HA variant 4 (C136S) (green color); (**F**) Structure of HA variant 5 (L227P) (white color). (**G**) Structure of HA variant 6 with all mutations of interest (C156S, D158N, Y159N, C136S, and L227P) (purple color). The red circle in all subfigures represent of active receptor-binding site (RBS). The structure in light pink in all subfigures is 1HGE.

According to the Ramachandran analysis, more than 95% of the amino acid residues in the five HA models were located in the most favored regions (*ψ* and *ϕ* angles). However, two specific residues (Val196 and Pro95) were observed in unfavorable regions within the protein structure, accounting for 0.4% of the total residues. It is important to note that, despite prior refinement for structural corrections, these stereochemical penalties originate from the template crystal structure (PDB ID: 1HGE) and were consequently carried over to the generated models (Figure 4).

These structural penalties did not adversely affect the *G*-factors, which confirm that the residue distribution is stereochemically normal. These results indicate that the obtained models are well-ordered and exhibit high structural quality (Supplementary Table S2, Supplementary Figure S3).

### 2.5. Molecular Docking Ligand-HA Variants

Molecular interactions between sialic acid and the HA models showed differences in binding affinity compared to the reference structure reported by Sauter et al. in 1992 (1HGE: −4.9 kcal/mol) [14].

A decrease to −4.5 kcal/mol is noted in V1-C156S and V5-L277P, followed by V2-D158N (−4.6 kcal/mol), V3-Y159N, V4-C136S, and V6-all (−4.7 kcal/mol). These differences are mediated by the substitutions of amino acid residues C156 with S, L227 with P, D158 with N, Y159 with N, and C136 with S, respectively. The key amino acids that actively participate in the formation of the HA-sialic acid complex through polar bonds (H-bonds) in the 1GHE crystal structure are Thr187, Glu190, Ser186, and Ser227 (Table 2).

**Figure 5:**
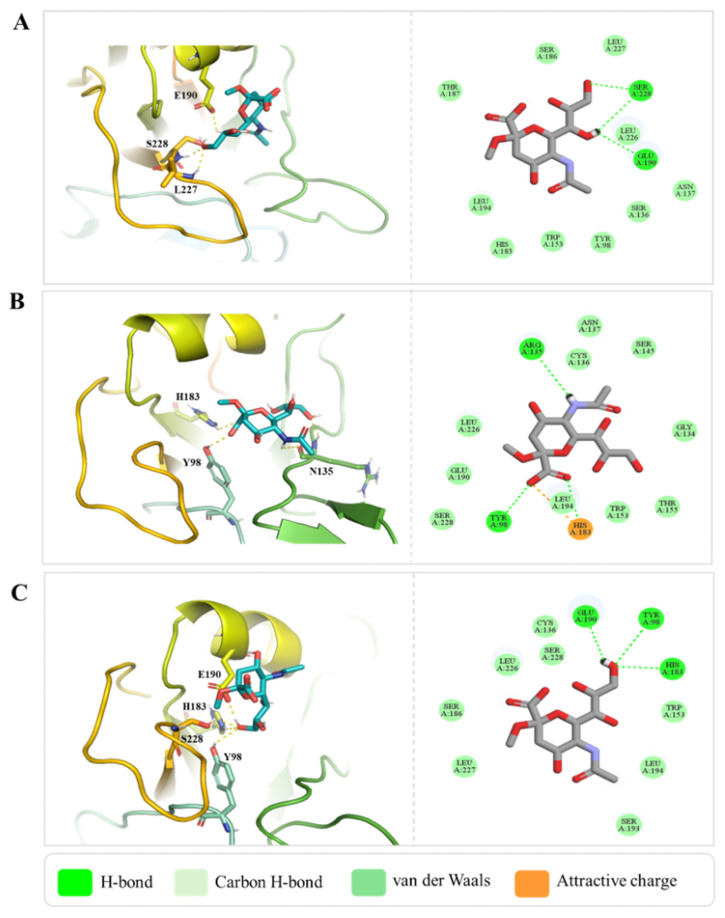
Molecular interactions between HA variants (V1, V2, and V3) and sialic acid in 3D and 2D representations. (**A**) Molecular interactions in V1-C156S; (**B**) Molecular interactions in V2-D158N; (**C**) Molecular interactions in V3-Y159N. The panels on the left show 3D representations of the molecular interactions between HA and the sialic acid molecule (blue). The yellow dotted lines represent the H-bond interactions of the sialic acid and the participating amino acids. The panels on the right show 2D representations of the molecular interactions; the circles indicate the amino acids, and the color refers to the type of interaction.

**Figure 6:**
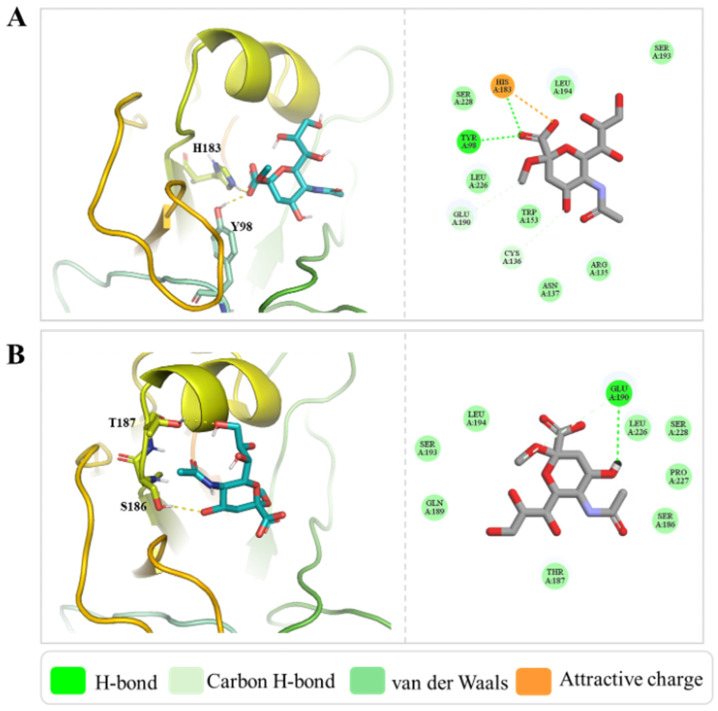
Molecular interactions between HA variants (V4 and V5) and sialic acid in 3D and 2D representations. (**A**) Molecular interactions in V4-C136S; (**B**) Molecular interactions in V5-L227P. The left panels show 3D representations of the molecular interactions between HA and the sialic acid molecule (blue). The yellow dotted lines represent the H-bond interactions of the sialic acid and the participating amino acids. The panels on the right show 2D representations of the molecular interactions; the circles indicate the amino acids, and the color refers to the type of interaction.

**Figure 7:**
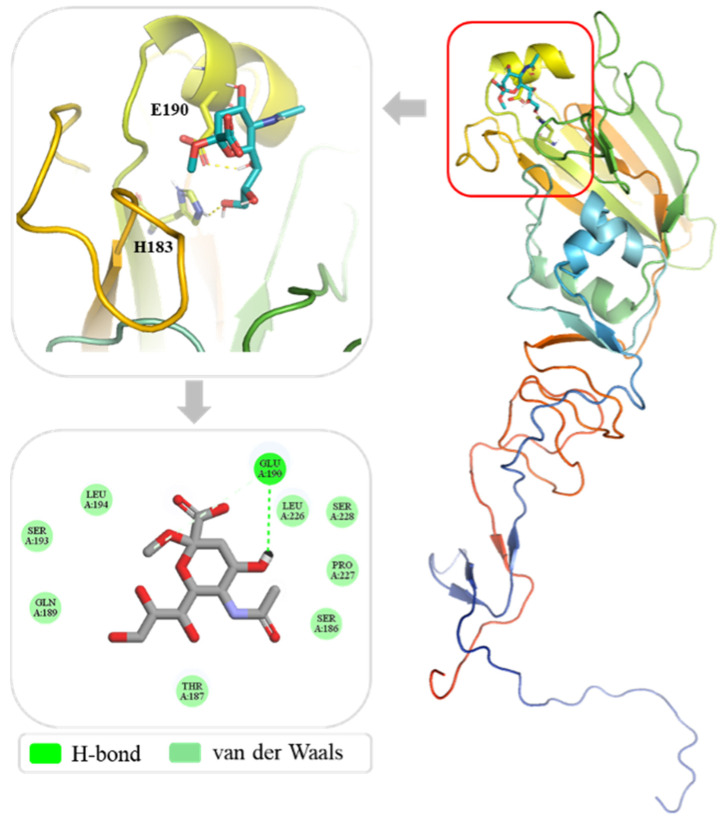
Molecular interactions between V6-all HA and sialic acid in 3D and 2D representations. The upper left panel shows a 3D representation of the molecular interactions in close-up, where the yellow lines represent H-bond interactions with the sialic acid molecule shown in blue. The lower left panel shows a 2D representation of the molecular interactions; the circles indicate the amino acids, and the color refers to the type of interaction.

The complexes formed between the HA variants and sialic acid exhibit conserved interactions despite the point mutations present in each variant. Of the four bonds observed in the HA/sialic acid complex (1HGE), the bond established by Glu190 is conserved; this residue participates in hydrogen-bond interactions in the V1-C156S, V3-Y159N, and V6-all complexes, while in the V2-D158N and V4-C136S complexes, this amino acid residue interacts noncovalently, stabilizing the ligand (sialic acid) binding. In contrast to the crystallized HA/sialic acid complex and the V5-L227P complex, the V1-C156S, V2-D158N, V3-Y159N, V4-C136S and V6-all complexes showed consistent involvement of the His183 residue, establishing both strong bonds (H-bonds) and weak bonds, primarily Van der Waals forces and attractive charges (Figure 5, Figure 6 and Figure 7).

## 3. Discussion

During the 2022–2023 period, A(H3N2) was the dominant flu strain in Mexico, representing 84.3% of reported cases, according to the Mexican General Directorate of Epidemiology [15]. Our study reflected a similar pattern, with A(H3N2) accounting for 88% of positive flu cases. No significant differences were observed between genders in the distribution of A(H3N2) and non-subtypeable influenza A (*p* = 0.391). The only detected case of influenza B lineage Victoria occurred in a female patient. The highest incidence was recorded in the 20–24 and 25–29 age groups, mirroring national surveillance trends [15]. This predominance among young adults may reflect greater social interaction, occupational and academic exposure, and increased mobility, all of which favor influenza virus transmission. In contrast, influenza cases among individuals over 65 years of age were notably lower, likely due to the location of the diagnostic laboratory, where younger populations have greater access to testing. Additionally, older adults are more likely to seek care, potentially leading to their underrepresentation in our dataset. These findings should therefore be interpreted in the context of the study population and healthcare access patterns rather than as a direct reflection of the age-specific burden of influenza in the general population. The peak of infections in our study occurred in weeks 1, 46, and 45, aligning with the early weeks of the 2022–2023 flu season. Studies have shown that A(H1N1) and A(H3N2) flu viruses spread similarly, influenced by factors like globalization and air travel, with a faster transmission in winter, regardless of population density. This may explain the similarity between the strains identified in our study and those circulating in the United States during the same period. The high mobility of people between these neighboring countries likely contributes to this pattern [16,17].

Additionally, the epidemiological weeks with the highest number of cases (weeks 45 to 48) coincide with the onset of colder temperatures in November, creating favorable conditions for virus transmission [18].

Regarding the genetic diversity of influenza viruses, sequence variation occurs throughout the eight viral RNA segments. The *HA* gene, encoded by segment 4, has been extensively investigated owing to its central role in viral classification and molecular surveillance [19].

Studies suggest that for the virus to sufficiently alter its antigenicity and form a new strain, at least four amino acid changes must occur, affecting two or more of the five antigenic sites (A, B, C, D, E) [20].

However, Koel et al. proposed that changes in just seven specific amino acid positions (145, 155, 156, 158, 159, 189, and 193) are primarily responsible for the major antigenic shifts in the virus. These positions are on the periphery of the RBD, and even a single variation at any of them can lead to the emergence of a new viral cluster [21].

Regarding our study, 60.9% (*n* = 39) of the identified amino acid substitutions were located within the RBD, highlighting this region as a major site of genetic variation among the detected HA variants. Given the established role of the RBD in receptor recognition and antigenicity, these findings support its relevance for molecular surveillance and warrant further functional investigation. These changes affect the virus’ affinity for sialic acid in the respiratory tract. Notably, 14 of the 39 variants identified within the RBD subdomain were concentrated around amino acid position 200 (±10 positions), including variants such as F192I, P197Q, V200G, F200G, K201R, E201R, F202I, S203T, F205S, T207K, K208R, G208R, R209S, and R210Q. Additionally, the S200P variant, which was once rare but plays a crucial role in binding to sialic acid, has been increasing in prevalence in recent years. By the end of 2019, its prevalence had risen from 28.13% to 93.51% in the United States and Europe [22].

This variant induces structural alterations in the RBD, resulting in functional modifications and influencing the production of antibodies targeting HA [23]. Replacing proline with any other amino acid at this position will directly affect the secondary structure and folding, enhancing the virus’s avidity and transmissibility [24].

Here, we identified the V200G variant, in which glycine is replaced by valine, and the F200G variant, in which glycine is replaced by phenylalanine at the same position. In both instances, these changes involve substituting one nonpolar amino acid for another of the same type, suggesting they may not lead to significant structural or functional alterations in HA. We also identified variants in other HA1 subdomains.

In subdomain E, variants at position 276 were notable. These included amino acid substitution K276R, E276K, K276R, and Q276K, where the replacement of arginine or lysine (positively charged hydrophilic amino acids) by glutamic acid (a negatively charged hydrophilic amino acid) or glutamine (a polar hydrophilic amino acid) was predominant. This position, found in the HA stem, has been described as a potential glycosylation site for swine flu A(H1N1) strains circulating in Japan from 2013 to 2018, although not for H3N2 strains. These changes could be significant in increasing the virus antigenicity [25].

We also detected six variants in the F’ portion of the HA: E286G, S289P, R303G, K307R, A309V, and E315K. In comparison, circulating strains from Russia exhibited variants such as K283E, A315V, and I321V, which were present in most strains and are located at non-antigenic sites [26].

Although, HA2 consists of a highly conserved stem-like region that links the globular domain to the viral membrane and contains the viral fusion peptide [27]. In our findings, this region appears largely conserved; however, we observed some variants, primarily concentrated in the F-HR fusion peptide region and the TM transmembrane region. While variants in HA2 are not extensively documented, Kuzmanovska et al. reported some substitutions in this subunit of HA, such as G155E, G150E, and G484E in influenza A(H3N2), and V192A, I76M, N169S, and E179D in AH1N1, respectively [28]. Among these variants, the G484E substitution has been shown to potentially have a negative impact on the function of HA2 [29].

The RBD is crucial for antigenic variation because it plays an active role in the virus’s interaction with sialic acid-containing receptors on the host cell, leading to virion internal-ization and the progression of the infection. Reports analyzing the bonds in the A(H3N2) HA/sialic acid complex (PDB: 4GZQ) report an affinity energy of −5.9 kcal/mol, highlighting polar interactions with residues Glu227, Arg152, Arg292, His183, Tyr98, Asp190, and Gln226 [30,31].

As in this study, we observed a binding affinity of −4.9 kcal/mol, and the residues involved in binding were Ser186, Thr187, Glu190, and Ser227. These residues are located within or near the RBD and are important for the stability of HA-sialic acid binding in H3N2 influenza A viruses [11].

The mutation rate in influenza viruses varies depending on the host species; some mutations are synonymous, while others are non-synonymous. Accumulation of amino acid substitutions in the HA protein contributes to its genetic and antigenic diversity. Previous studies have shown that specific substitutions may alter receptor-binding properties or antigenic characteristics, although their biological consequences depend on the identity and combination of the mutations involved and require experimental validation [32,33,34]. In this study, the high variability of mutations found in the HA of patients showed that the most relevant predicted interactions involved the C156S, Y159N, C136S and L227P (3.12%), and D158N (6.25%) variants. These mutations are located at or near the sialic acid receptor-binding site of A(H3N2) HA, which is key to viral interaction with host cells and to viral success in infecting host cells [35].

While some of these mutations may have a significant structural impact, such as C156S and C136S, which result in the loss of disulfide bonds, or L227P, which replaces a flexible amino acid with a rigid, hydrophobic one, this mutation may cause breaks in the HA alpha helices and may affect binding to the host. Previous studies have suggested that mutations in HA can also confer advantages to the virus for its spread, such as the removal of glycosylation sites, which may contribute to reduced immune recognition [34,36,37]. Specifically, interaction with the 190-helix region located in the RBD is critical for host specificity and binding to sialic acid receptors [13,38].

In all analyzed HA variants, Glu190 was consistently involved in the predicted HA–sialic acid interaction, through H-bonds (V1-C156S, V3-Y159N, and V5-L227P) and non-covalent bonds (V2-D158N and V4-C136S), suggesting preservation of key structural interactions within the modeled HA–sialic acid complexes despite the analyzed substitutions. The C136S mutation, which accounts for 3.12% of all mutations in the RBD region, may induce local structural changes with potential effects on receptor binding and immune recognition; however, these potential effects require experimental validation. On the other hand, the D158N and Y159N mutations have been described as high-impact changes in host binding, as they can completely reshape molecular interactions by promoting binding flexibility of the A(H3N2) HA [13]. Consistent with earlier studies, the complexes with variants V2-D158N and V3-Y159N displayed changes in the predicted interaction network where the residues involved in complex formation were Tyr98, Arg135, and His183, and Tyr98, His183, Ser228, and Glu190, respectively. These changes were associated with slight differences in the predicted binding affinity of V2-D158N to −4.6 and of V3-Y159N to −4.7 kcal/mol. Both Tyr98 and His183 play important roles in the stability of the RBD region, which enables proper binding to sialic acid [39,40].

Several studies have reported the crucial role of the His183 residue, which is highly conserved in influenza A viruses and has been essential following the species jump [11]. Several amino acid substitutions have been described in the HA protein of recently identified clade 2a.3a.1 variants, particularly within antigenically relevant regions, whereas His183 has remained conserved [41]. Consistent with this observation, our docking analyses identified His183 as a recurrent residue involved in the predicted HA–sialic acid interaction across all analyzed variants, highlighting its potential structural contribution to receptor recognition despite sequence variation in adjacent regions.

### Limitations and Strengths

This study provides a molecular and structural characterization of Influenza A(H3N2) hemagglutinin. However, this work has some limitations that should be considered. First, the dataset was limited to samples collected through surveillance conducted at a single diagnostic laboratory in a single geographic region in Western Mexico, which may limit the generalizability of the findings to other regions with different epidemiological dynamics. Furthermore, although a considerable number of cases were analyzed, only a subset of viral isolates underwent HA sequencing and structural modeling, which limited the assessment of genetic variation in other genetic segments of the influenza virus, such as NA mutations, internal genetic mutations, complete genomic relationships, or rearrangements. This could have underestimated the overall genetic diversity and the occurrence of genomic rearrangements among circulating strains. Likewise, no functional in vitro or in vivo assays were performed to experimentally validate the effects of the identified mutations on viral fitness, transmissibility, or immune evasion; thus, interpretations of their biological relevance rely primarily on computational predictions. In addition, the molecular docking analyses provide static structural predictions of receptor–ligand interactions and do not account for protein conformational flexibility, solvent effects, or dynamic molecular behavior, which may influence binding interactions under physiological conditions. Additionally, although Sanger sequencing is a robust and reliable method, it may have limited sensitivity for detecting low-frequency variants compared with next-generation sequencing technologies. Finally, the limited integration of clinical data precluded a more comprehensive evaluation of genotype–phenotype associations.

Despite these limitations, this study presents several notable strengths. It provided molecular evidence regarding Influenza A(H3N2) HA variants circulating during the 2022 epidemiological year, thereby contributing to a better understanding of temporal circulation patterns in a region with limited genomic surveillance data. The integration of epidemiological, genetic, and structural analyses represents a key strength, allowing for a multidimensional understanding of viral evolution. The identification of mutations concentrated within the receptor-binding domain (RBD), coupled with homology modeling and molecular docking, provides structural insights into the potential effects of these substitutions on HA–receptor interactions. Furthermore, the study highlights conserved residues involved in the predicted receptor interaction, suggesting conservation of key structural interaction patterns across the analyzed variants. These findings provide complementary molecular information that supports regional genomic surveillance strategies, establishes a basis for future functional and antigenic studies, and may provide information for pandemic preparedness efforts.

## 4. Materials and Methods

### 4.1. Study Design and Participants

This retrospective study included a total of 476 participants from Western Mexico who tested positive for the influenza virus via nasopharyngeal swabs. The samples were analyzed at the Laboratorio de Diagnóstico de Enfermedades Emergentes y Reemergentes (LaDEER), Centro Universitario de Ciencias de la Salud of Guadalajara, Mexico, during the period from 1 January to 31 December 2022.

### 4.2. Molecular Diagnosis and Subtyping

Viral RNA was isolated from nasopharyngeal swabs stored in a viral transport medium using the Quick-RNA Viral Kit (Zymo Research, Irvine, CA, USA). Flu virus detection was performed using qRT-PCR with the CoviFlu Kit Multiplex (Genes2Life, Mexico City, Mexico). Samples were considered positive when viral genes were detected with a cycle threshold value *CT* < 35, while the absence of viral genes indicated a negative result. PCR analyses were performed using the QuantStudio™ 5 Real-Time PCR System (Applied Biosystems, Waltham, MA, USA). Subtyping was performed by RT-qPCR using the BlueFinder™ 22 kit (Genes2Life, Mexico City, Mexico), identifying flu A(H1N1) 09pdm, A(H3N2) (seasonal), and B (Victoria and Yamagata).

### 4.3. Variant Identification

Viral RNA was reverse transcribed into complementary DNA (cDNA) using the Verso cDNA Synthesis Kit (Thermo Fisher Scientific, Waltham, MA, USA), following the manufacturer’s protocol. Specific PCR primers were used instead of random hexamers. The reaction was performed at 42 °C for 30 min, followed by inactivation at 95 °C for 2 min.

Amplification of the complete HA gene (1762 bp) was performed using the Q5^®^ High-Fidelity DNA Polymerase kit (New England Biolabs, Ipswich, MA, USA) with specific primers (Forward: 5′-AGCAAAAGCAGGGGATAATTC-3′ and Reverse: 5′-AGTAGAAACAAGGGTGTTTTTA-3′). Reactions were carried out in an Applied Biosystems thermal cycler. The PCR conditions consisted of an initial denaturation at 98 °C for 2 min, followed by 35 cycles of 30 s at 98 °C, 60 s at 59 °C, and 2 min at 72 °C, with a final extension at 72 °C for 10 min. All reactions were performed in duplicate and verified by 1% agarose gel electrophoresis, following the methodology described by Dinis et al. [42].

### 4.4. Purification and Sanger Sequencing

PCR products were purified using the Wizard^®^ SV Gel and PCR Clean-Up System (Promega Corporation, Madison, WI, USA) and quantified using the Qubit™ fluorometer (Thermo Fisher Scientific, Waltham, MA, USA). The HA gene was sequenced bidirectionally using the Sanger method on Genetic Analyzer 3500 and 3130 platforms (Applied Biosystems, Waltham, MA, USA). Sequencing was performed through the service of the Laboratorio Nacional de Biotecnología Agrícola, Médica y Ambiental (LANBAMA) del Instituto Potosino de Investigación Científica y Tecnológica (IPICYT), following a protocol adapted from Lee et al. [43].

### 4.5. Sequence Assembly and Similarity Analysis

Pairwise nucleotide sequence alignments were performed using the EMBOSS Needle software [44] for the assembly of the forward and reverse sequences of the HA gene. Following assembly, a similarity analysis was conducted using the Basic Local Alignment Search Tool (BLASTn 2.17.0+) to compare the obtained sequences with those reported in the National Center for Biotechnology Information (NCBI) database. Comparisons focused on strains circulating during the 2022–2023 period in Mexico and other North American regions, utilizing data retrieved from the NCBI Influenza Virus Database.

### 4.6. Phylogenetic Analysis and Clade Assignment

Phylogenetic analysis was performed using Nextclade version 3.21.1 [45] to assign clades and subclades based on the HA segments. A dataset of reference HA sequences was constructed from the GISAID database to provide context for the circulating subclades. This approach allowed for the identification of specific mutational profiles and the classification of variants according to the current global nomenclature for influenza A(H3N2). The resulting phylogenetic tree was visualized and annotated using the Interactive Tree of Life (iTOL) version 7 [46].

### 4.7. Protein Translation and Multiple Sequence Alignment

Nucleotide sequences were translated into amino acid sequences using the ExPASy Translate tool [44]. Multiple sequence alignments of the resulting amino acid sequences were performed using the MUSCLE algorithm [47]. To evaluate amino acid substitutions and map mutations, the A/Darwin/6/2021 vaccine strain (recommended for the 2022–2023 Northern Hemisphere season) along with sequences circulating in the North American region during 2022, were included in the analysis as reference sequences for variant comparison [48].

### 4.8. Structural Modeling and Validation of HA Variants

Five HA variants were selected based on their antigenic and functional relevance, specifically focusing on amino acid substitutions within the RBD [11], in addition to being the most representative in the study population (Supplementary Table S1). The corresponding amino acid sequences were retrieved from GenBank (accession numbers QNS17499.1 and BAA04708.1). The crystal structure of the H3N2 hemagglutinin (PDB ID: 1HGE, resolution 2.60 Å) was used as a template to establish the molecular interactions of the binding site in the RBD [14].

Homology modeling was performed using MODELLER v8 [49,50] to generate three dimensional structural models for the five selected variants. The resulting structures were subsequently refined using the GalaxyWEB server [51]. To ensure the reliability of the models, structural quality was validated using the SAVES v6.1 server [52], which included the following indices: ERRAT [53], VERIFY 3D [54], Ramachandran plot analysis [55], MolProbity [56], ProSA [57], and QMEAN [58].

### 4.9. Molecular Docking Analysis

The ligand, a single sialic acid molecule co-crystallized in the HA structure (PDB ID: 1HGE), served as the structural guide to identify the binding site [14]. The 3D models of the HA variants and the ligand were prepared by removing crystallographic waters, adding polar hydrogens, and assigning atomic charges. Specifically, Kollman (AM1-BCC) charges were assigned to the HA variants, while Gasteiger charges were assigned to the ligand. Files were converted to PDBQT format using UCSF Chimera v1.5 [59] and AutoDock Tools v1.5.6 [60]. The grid box was focused on the RBS, centered at coordinates *x* = −4.35, *y* = 71.0, and *z* = 27.0, with a box size of 20 × 20 × 20 Å. To validate the docking protocol, a redocking analysis was performed using AutoDock Vina v1.1.2 [61]. The RMSD between the experimentally determined ligand conformation and the best-ranked docking pose was calculated using UCSF Chimera v1.5 [59]. An RMSD value of 1.133 Å was obtained, which is below the 2.0 Å threshold, supporting the reliability of the docking methodology (Supplementary Figure S1). For each HA variant, 100 independent docking simulations were performed using AutoDock Vina to ensure that the search converged to a consistent binding mode. The simulations were run using a semi-flexible model (rigid receptor and flexible ligand) with a thoroughness level set to 8. The best-scoring pose for each variant, defined by the lowest binding free energy (Δ*G* in kcal/mol), was selected for further analysis. Hydrogen bonds and hydrophobic contacts were analyzed and visualized using PyMOL v. 3.1.6.1 [62] and Discovery Studio v. 2021 [63]. Binding affinities and interaction patterns of the five HA variants were compared with the wild-type structure to assess changes in key residues and the stability of the ligand–HA complex [61,64,65,66].

### 4.10. Statistical Analysis

This study was designed as a descriptive molecular surveillance analysis; therefore, descriptive statistics were used to characterize the epidemiological and molecular features of the dataset. Categorical variables, including viral subtype distribution, HA amino acid substitutions, genetic variant frequencies, clade assignment, epidemiological week (EW), and demographic characteristics, were summarized as frequencies and percentages. Continuous variables were reported as mean ± standard deviation for normally distributed data. The distribution of HA genetic variants was evaluated according to temporal occurrence, demographic characteristics, and phylogenetic classification to describe patterns of circulating Influenza A(H3N2) diversity. Comparisons of categorical variables, including viral subtype distribution according to sex and age groups, were performed using the Chi-square test when applicable. A *p*-value < 0.05 was considered statistically significant. Statistical analyses were performed using IBM SPSS Statistics for Windows, version 27.0 (IBM Corp., Armonk, NY, USA).

## 5. Conclusions

This study provides molecular and structural evidence regarding Influenza A(H3N2) hemagglutinin variants circulating in Western Mexico during 2022, identifying sequence diversity, with most amino acid substitutions mapped to the receptor-binding domain (RBD). Despite this variability, residues involved in the predicted HA–sialic acid interaction remained conserved across in analyzed sequences, suggesting preservation of key structural features associated with receptor recognition. These findings support the integration of genomic surveillance with structural and epidemiological analyses to improve the molecular characterization of circulating A(H3N2) variants and provide complementary information for public health surveillance.

## Figures and Tables

**Table 1:** Quality scores of HA models.

Models	ERRAT (%)	Verify 3D (%)	QMEAN	MolProbity	ProSA (Z)	ProQ (LG)
V1-C156S	90.81	68.60	−0.38	1.45	−7.06	10.88
V2-D158N	91.21	67.38	−0.47	1.47	−6.80	11.08
V3-Y159N	87.24	71.04	−0.76	1.55	−6.79	11.14
V4-C136S	90.81	69.21	−0.31	1.44	−7.19	11.11
V5-L227P	91.21	69.82	−0.49	1.39	−6.88	11.13
V6-all	92.63	71.04	−0.57	1.37	−7.09	11.01

**Table 2:** Molecular docking results: binding affinities and molecular interactions between sialic acid and the HA models.

Models	Affinity (kcal/mol)	Polar Bonds	Distances	Non-Covalent Interactions
1HGE	−4.9	Thr187–O, Glu190–H, Ser186–O, Ser227–O	2.5, 2.4, 2.0, 2.2	Gln189, Leu194, Ser228, Leu226, Trp222, Gly225
V1-C156S	−4.5	Glu190–H, Ser228–O, Leu227–O	2.3, 2.0, 2.3	Leu227, Leu226, Asn137, Ser136, Tyr98, Trp153, His183, Leu194, Thr187, Ser186
V2-D158N	−4.6	His183–O, Tyr98–O, Arg135–H, Arg135–O	2.4, 2.2, 2.7, 2.3	Cys136, Asn137, Ser145, Gly134, Thr155, Trp153, Leu194, His183, Ser228, Glu190, Leu226
V3-Y159N	−4.7	Glu190–H, His183–O, Tyr98–O, Ser228–O	2.0, 2.1, 2.6, 2.3	Trp153, Leu194, Ser193, Leu227, Ser186, Leu226, Ser228, Cys136
V4-C136S	−4.7	Tyr98–O, His183–O	2.1, 2.2	Leu194, Ser193, Arg135, Asn137, Trp153, Cys136, Glu190, Leu226,
				Ser228, His183
V5-L227P	−4.5	Thr187–O, Ser186–O	2.3, 2.5	Glu190, Leu226, Ser228, Pro227, Ser186, Thr187, Gln189, Ser193,
				Leu194
V6-all	−4.7	Glu190–H, His183–O	2.1, 2.4	Leu226, Ser228, Pro227, Trp153, Glu190, Leu194, Gln189, Ser193,
				Thr187, Ser186

## Data Availability

The original contributions of this study are included in the article and Supplementary Materials. For additional inquiries, contact the corresponding author. The partial nucleotide sequences generated and analyzed during the current study have been deposited in the NCBI GenBank database under the accession numbers PZ638017 to PZ638028. For additional inquiries, contact the corresponding author.

## Supplementary Materials

The following supporting information can be downloaded at: https://www.mdpi.com/article/10.3390/ijms27156833/s1.
