# Supplementary material for: Molecular Characterization of Influenza A(H3N2) Hemagglutinin Variants Circulating in Western Mexico, 2022
Source: Int J Mol Sci. 2026 Jul 30;27(15):6833. doi: 10.3390/ijms27156833 (PMC13466227; doi:10.3390/ijms27156833)
Supplement: Supplementary file 1 [file ijms-27-06833-s001.zip › ijms-4425424-supplementary.pdf]

Supplementary Table S1. *Amino acid variants observed on the hemagglutinin protein of influenza virus H3N2 circulating in Western Mexico during 2022.*

Epidemiologic week	Variant	HA protein region	Subdominium
Week 04	I157L	HA1	RBD
	A175D	HA1	RBD
	K201R	HA1	RBD
	S203T	HA1	RBD
	R209S	HA1	RBD
	R210Q	HA1	RBD
Week 22	N262S	HA1	RBD
	K276R	HA1	E
	S289P	HA1	F´
	Y534F	HA2	TM
Week 23	Y126N	HA1	RBD
	M140I	HA1	RBD
	F192I	HA1	RBD
	F200G	HA1	RBD
	E201R	HA1	RBD
	F205S	HA1	RBD
	K276R	HA1	E
	S378N	HA2	F-HR
Week 24	Y126N	HA1	RBD
	C136S	HA1	RBD
	M140I	HA1	RBD
	D158N	HA1	RBD
	F192I	HA1	RBD
	K208R	HA1	RBD
	K276R	HA1	E
	E286G	HA1	F´
	S378N	HA2	F-HR
Week 27	G86R	HA1	E
	N262S	HA1	RBD
	E276K	HA1	E
	E404G	HA2	F-HR
	M452K	HA2	F-HR

Week 28	T141R	HA1	RBD
	P197Q	HA1	RBD
	Y524C	HA2	TM
	P527L	HA2	TM
Week 39	N238K	HA1	RBD
	K276R	HA1	E
Week 45	K140I	HA1	RBD
	H156S	HA1	RBD
	G208R	HA1	RBD
	L227P	HA1	RBD
	N262S	HA1	RBD
	K276R	HA1	E
	D275G	HA1	E
	R303G	HA1	F′
	K307R	HA1	F′
	A309V	HA1	F′
	E315K	HA1	F′
Week 46	K140I	HA1	RBD
	K141R	HA1	RBD
	T150R	HA1	RBD
	C156S	HA1	RBD
	D158N	HA1	RBD
	Y159N	HA1	RBD
	R503K	HA2	Link
Week 48	V200G	HA1	RBD
	F202I	HA1	RBD
	T207K	HA1	RBD
	R503K	HA2	Link
Week 50	S141R	HA1	RBD
	F244L	HA1	RBD
	Q276K	HA1	E
	M277C	HA1	E

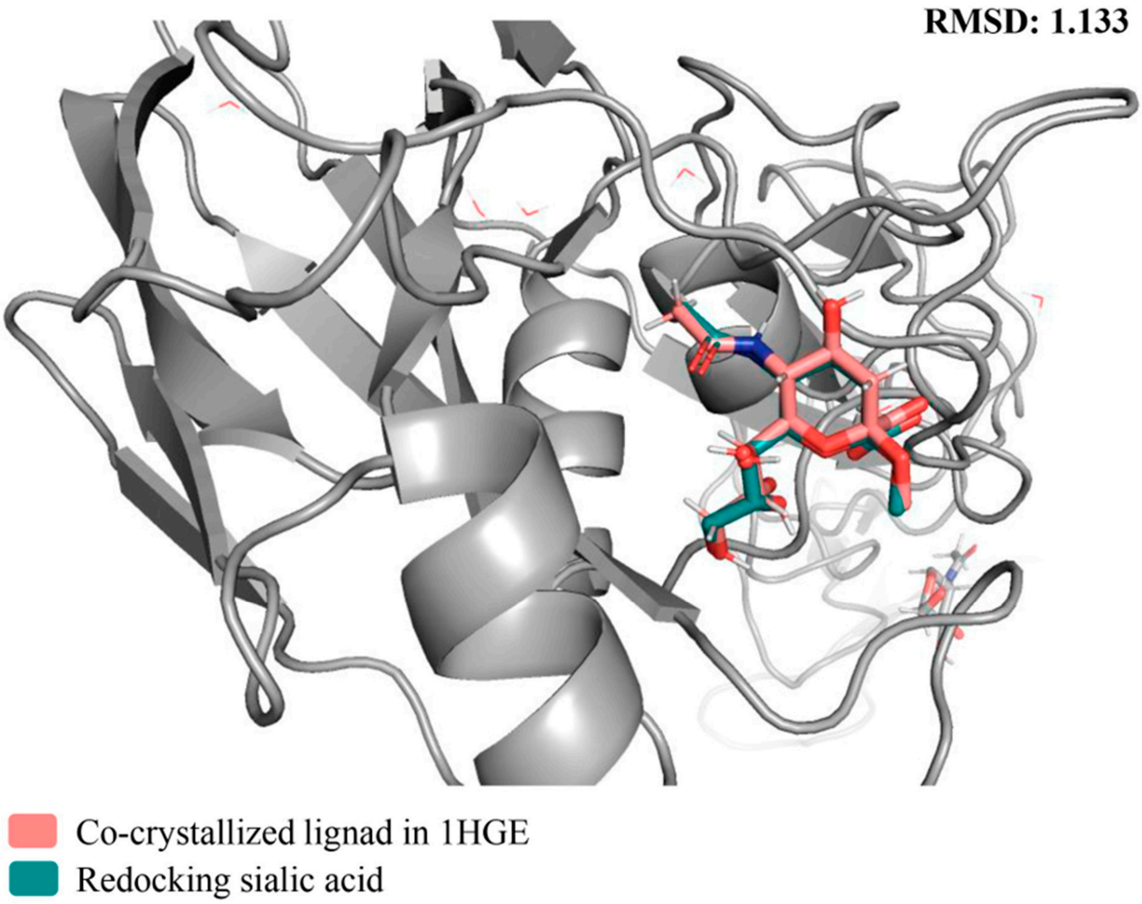

Supplementary Figure S1. *3D representation of redocking of crystallographic structures of the HA (1HGE) and its co-crystallized ligands. The PyMOL software was used.*

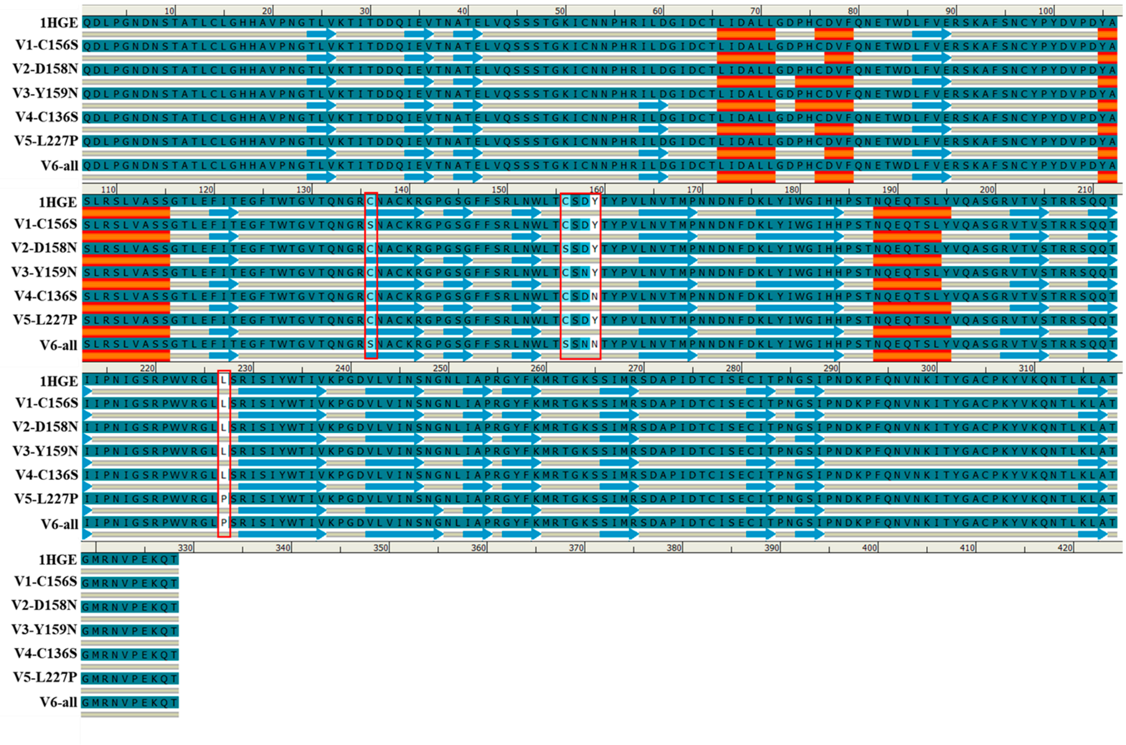

Supplementary Figure S2. *Sequence alignment of the reference, template, and target proteins used for modeling the five HA variants. The Discovery Studio software was used.*

Supplementary Table S2. *Ramachandran and G-factor values of HA models (PROCHECK in SAVE 6.0 server)*

	PROCHECK						
	Ramachandran plot				G-factor		
**Models**	**Most favored (%)**	**Additional allowed (%)**	**Generously allowed (%)**	**Disallowed (%)**	**Dihedral**	**Covalent**	**Overall**
V1-C156S	95.4	3.2	1.1	0.4	0.27	-0.25	0.08
V2-D158N	95.0	3.5	1.1	0.4	0.27	-0.27	0.07
V3-Y159N	94.7	3.9	1.1	0.4	0.25	-0.32	0.04
V4-C136S	95.7	3.2	0.7	0.4	0.27	-0.30	0.06
V5-L227P	94.3	4.3	1.1	0.4	0.26	-0.24	0.07
V6-all	96.1	2.5	1.1	0.4	0.29	-0.21	0.10

The quality indices confirmed that the protein structures of the five HA models are adequate, meaning they can be used in subsequent analyses such as molecular docking.

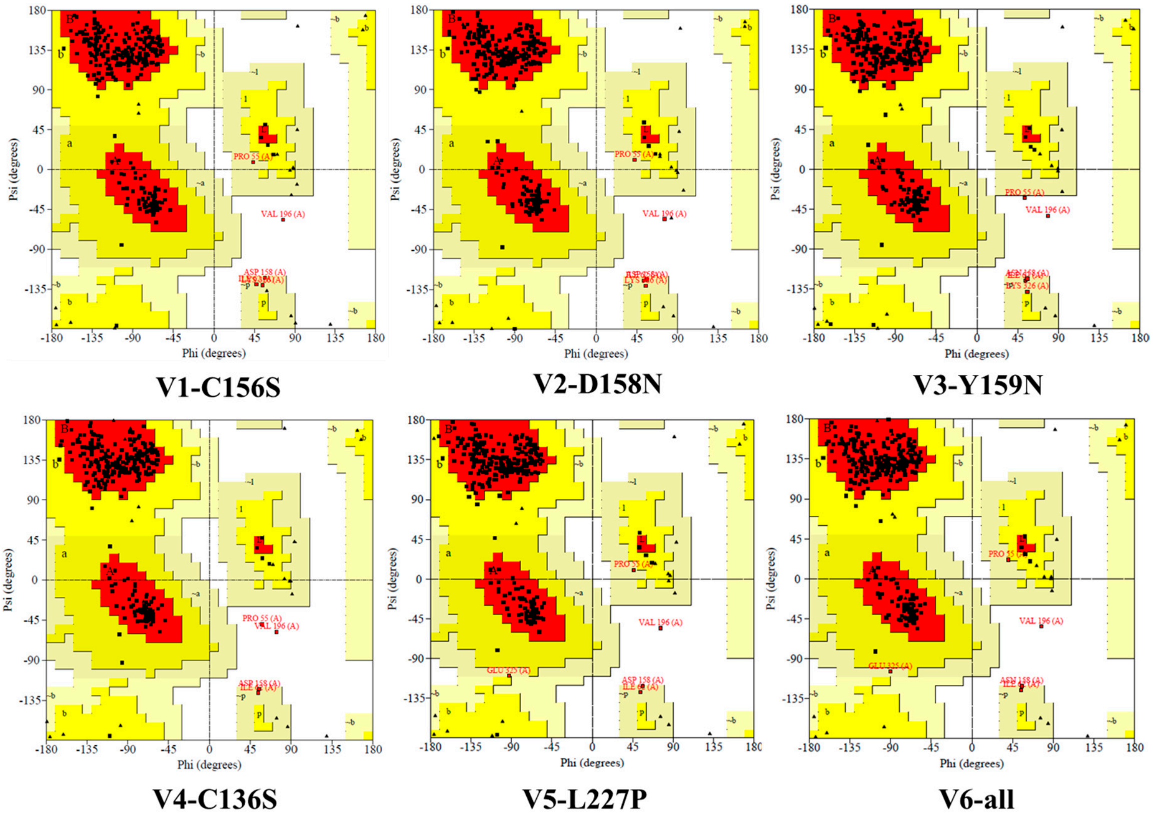

Supplementary Figure S3. *Ramachandran plots of the HA models (V1-C156S, V2-D158N, V3-Y159N, V4-C136S, V5-L227P, and V6-all. The red zone represents the most favored regions. The yellow zone represents the allowed regions. The pale yellow zone represents the generously allowed regions. The white zone represents the disallowed regions. Black dots represent the amino acid residues.*
